# Iodide of Amonium

**Published:** 1861-02

**Authors:** 


*Iodide of Amonium.*—This drug is reputed valuable in all cases where potas. iod. is indicated, particularly in constitutional syphilis. Its claimed advantages are : greater acceptability to the stomach, its effect more rapidly produces, and the quantity required less. From two to sixteen grains given per day.—*Cin. Lancet & Observer.*

